# Defective *Atg16l1* in intestinal epithelial cells links to altered fecal microbiota and metabolic shifts during pregnancy in mice

**DOI:** 10.1080/19490976.2024.2429267

**Published:** 2024-12-02

**Authors:** Víctor A. López-Agudelo, Maren Falk-Paulsen, Richa Bharti, Ateequr Rehman, Felix Sommer, Eike Matthias Wacker, David Ellinghaus, Anne Luzius, Laura Katharina Sievers, Manuel Liebeke, Arthur Kaser, Philip Rosenstiel

**Affiliations:** a Institute of Clinical Molecular Biology, Christian-Albrechts-University, and University Hospital Schleswig-Holstein, Kiel, Germany; b Boehringer Ingelheim, Biberach an der Riß, Germany; c DSM Nutritional Products, Kaiseraugst, Switzerland; d Department of General Internal Medicine, Christian-Albrechts-University and University Hospital Schleswig-Holstein, Kiel, Germany; e Department for Metabolomics, Institute for Human Nutrition and Food Science, University of Kiel, Kiel, Germany; f Department of Symbiosis, Max Planck Institute for Marine Microbiology, Bremen, Germany; g Cambridge Institute of Therapeutic Immunology and Infectious Disease (CITIID), Jeffrey Cheah Biomedical Centre, and Division of Gastroenterology and Hepatology, Department of Medicine, University of Cambridge, Cambridge, UK

**Keywords:** Pregnancy, microbiota, IBD, *Atg16l1*

## Abstract

Throughout gestation, the female body undergoes a series of transformations, including profound alterations in intestinal microbial communities. Changes gradually increase toward the end of pregnancy and comprise reduced α-diversity of microbial communities and an increased propensity for energy harvest. Despite the importance of the intestinal microbiota for the pathophysiology of inflammatory bowel diseases, very little is known about the relationship between these microbiota shifts and pregnancy-associated complications of the disease. Here, we explored the longitudinal dynamics of gut microbiota composition and functional potential during pregnancy and after lactation in *Atg16l1*^*∆IEC*^ mice carrying an intestinal epithelial deletion of the Crohn’s disease risk gene *Atg16l1*. Using 16S rRNA amplicon and shotgun metagenomic sequencing, we demonstrated divergent temporal shifts in microbial composition between *Atg16l1* wildtype and *Atg16l1*^*∆IEC*^ pregnant mice in trimester 3, which was validated in an independent experiment. Observed differences included microbial genera implicated in IBD such as *Lachnospiraceae*, *Roseburia*, *Ruminococcus*, and *Turicibacter*. Changes partially recovered after lactation. Additionally, metagenomic and metabolomic analyses suggest an increased capacity for chitin degradation, resulting in higher levels of free N-acetyl-glucosamine products in feces, alongside reduced glucose and myo-inositol levels in serum around the time of delivery. On the host side, we found that the immunological response of *Atg16l1*^*∆IEC*^ mice is characterized by higher colonic mRNA levels of TNFα and CXCL1 in trimester 3 and a lower weight of offspring at birth. Understanding pregnancy-dependent microbiome changes in the context of IBD may constitute the first step in the identification of fecal microbial biomarkers and microbiota-directed therapies that could help improve precision care for managing pregnancies in IBD patients.

## Introduction

Pregnancy is a transient physiological state characterized by significant alterations in the body’s physiology. These changes encompass hormonal fluctuations, substantial shifts in energy metabolism,^1–3^ as well as changes in the immunological network state to safeguard the developing fetus, thereby maximizing reproductive success. This immune tolerance during pregnancy is most pronounced at the feto-maternal interface and also has a systemic effect by rebalancing pro-inflammatory and anti-inflammatory influences also in other tissue compartments.^4^ Higher levels of regulatory immune cells, like regulatory T cells (Tregs) and myeloid-derived suppressor cells (MDSCs), are detectable in the placenta and other tissue compartments, coupled with an increase in soluble immunosuppressive factors such as IL-10 and TGF-β.^5,6^ It has been suggested that many of these processes are controlled by sex hormones, including progesterone and estrogen. Evidence has emerged suggesting that variations in the composition and function of intestinal microbiota are contributing to this physiological adaptation, e.g. by increasing energy harvest from nutrient intake.^7–15^ Reports have highlighted bacterial community shifts between the first and third trimesters, with third-trimester fecal microbiota exerting a subtle pro-inflammatory tone, which may contribute to higher adiposity and insulin insensitivity.^10,12,16^ Yet, the exact mechanisms and the impact of these changes on pregnancy-induced immune alterations are still unclear.^17^

Despite improved therapies, optimal clinical care for female patients with chronic inflammatory bowel disease during and after pregnancy remains a challenge. Data on disease course modulation by pregnancy in IBD are complex, with a significant burden resulting from pregnancy complications and relapses during or after pregnancy.^18–20^ Little is known about how sex hormone shifts during pregnancy may affect intestinal inflammation in IBD.^21^ Recent evidence has shown that 30% of patients with IBD will suffer from flaring disease in the first half year after delivery despite continued immunosuppressive therapy.^22^ Given the significant role of the intestinal microbiota for most facets of IBD, it is tempting to speculate that microbial shifts during pregnancy could also contribute to the heterogeneous outcome of pregnancy and disease course modulation around pregnancy and lactation. A human cohort study in 46 pregnant IBD patients showed a stable reduction of IL-10 and IL-5 levels but increased IL-8 and interferon (IFN)γ levels throughout gestation compared to pregnant healthy controls. The authors found correlations between individual fecal microbial taxa and serum cytokines, e.g. positive association between *Ruminococcus* and IL-5 levels.^23^ A larger study of 358 pregnant IBD patients from Mount Sinai suggested a correlation of specific bacterial taxa with maternal fecal calprotectin levels^24^; however, no studies exist that causally link specific changes of the gut microbiome during pregnancy to inflammatory flares in IBD or vice versa IBD-related changes in intestinal microbes to pregnancy outcomes.

In the past decade, >200 IBD risk loci have been identified by genome-wide association studies and exome sequencing,^25^ that cluster in distinct molecular pathways, including autophagy,^26^ ER stress signaling, and innate immune sensing.^25,27^ Loss-of-function variants in autophagy genes, such as *ATG16L1*, only affect CD patients and have been associated with clear functional defects in intestinal epithelial cells (IECs), e.g., a Paneth cell deficiency.^28^ Inadequate autophagy mediated by ATG16L1, via the CD T300A risk allele, increases the vulnerability of epithelial cells to inflammation induced by bacteria and viruses.^29,30^ *Atg16l1* deficiency in the intestinal epithelium of mice leads to an age-dependent onset of subtle inflammatory changes in the ileal mucosa,^31^ as well as to a paradoxical inflammatory response to the normally pro-regenerative cytokine IL-22.^32^ Autophagy is intricately connected to the unfolded protein response (UPR) originating from the endoplasmic reticulum.^33^ The significance of this interplay is underscored by the discovery that mice with a dual deficiency in the UPR transcription factor gene *Xbp1* and *Atg16l1* in the intestinal epithelium exhibit spontaneous transmural and fistulizing ileal inflammations resembling human Crohn’s disease.^34^ Despite subtle findings, such as a transmissible susceptibility to DSS-induced colitis in mice with an IEC-specific deletion of the gene,^31^ the role of *Atg16l1* in regulating the microbiome remains unclear.

We here hypothesized that the dysfunction of *Atg16l1* in IECs – due to its altered anti-microbial effector function – could perturb the normal shift of the fecal microbiota normally observed during pregnancy. We speculated that the intestinal microbiota changes during and after pregnancy could modulate susceptibility to intestinal inflammatory responses and modulate pregnancy outcomes. In this longitudinal study, we investigated the fecal microbial and physiological responses to pregnancy in mice carrying an *Atg16l1*-deletion in the intestinal epithelium and their corresponding littermate controls. We analyzed the temporal dynamics of the microbiota through 16S rRNA gene profiling and shotgun metagenomics, aiming to correlate these findings with observed microbial and immunological responses in the host. Our study provides a comprehensive view on changes in microbial diversity during and after pregnancy. Notably, we highlight the shifting microbial composition and metabolic potential across pregnancy phases in *Atg16l1*^*fl/fl*^ and *Atg16l1*^∆IEC^ mice.

## Materials and methods

### Generation and housing of conditional knockout mice

**Figure 1.**
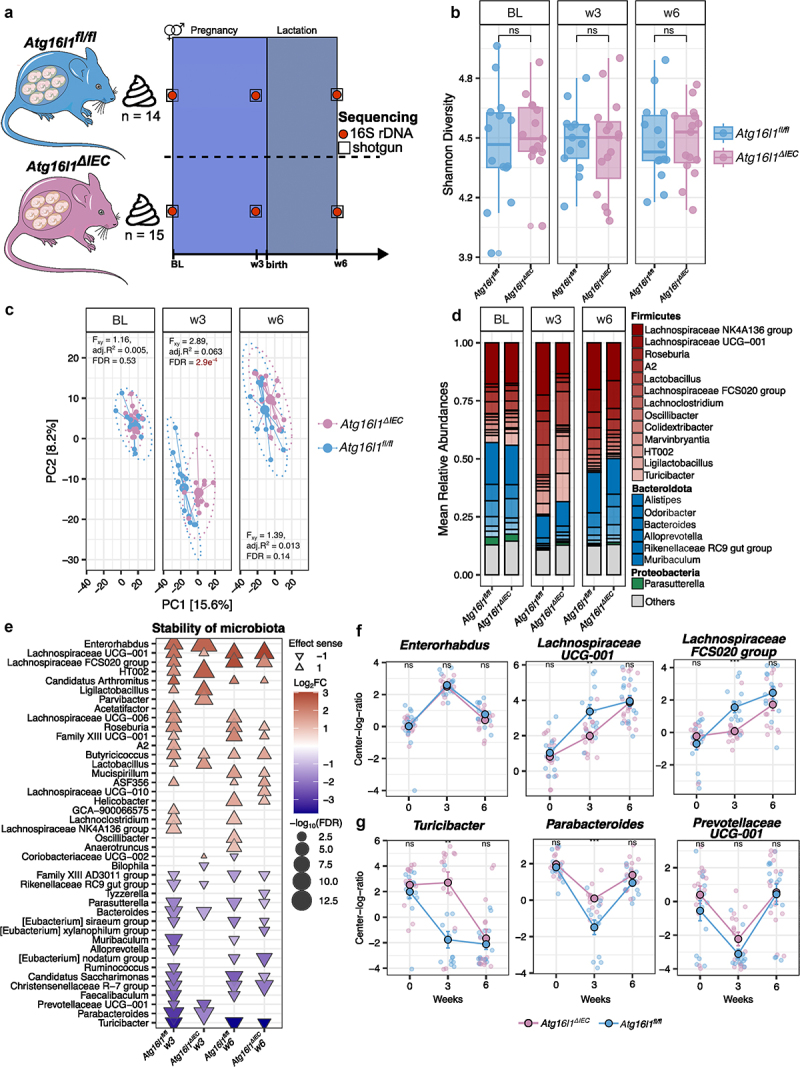
Gut microbiota characterization of *Atg16l1*^*fl/fl*^ and *Atg16l1*^*∆IEC*^ mice during pregnancy and after lactation periods. a. study design. Stool samples of *Atg16l1*^*fl/fl*^ (*n* = 14 mice) and *Atg16l1*^*∆IEC*^ (*n* = 15 mice) pregnant mice were collected at baseline (before mating), week 3 (late pregnancy), and week 6 (after lactation period). Samples were submitted to 16S rRNA and shotgun sequencing. b. alpha diversity analysis of gut microbiota during pregnancy and lactation. Shannon diversity was computed at the ASV level. Comparisons between *Atg16l1*^*fl/fl*^ (*n* = 14 mice, three timepoints) and *Atg16l1*^*∆IEC*^ (*n* = 15 mice, three timepoints) were performed using the Wilcoxon rank-sum test. c. principal coordinate analysis on Aitchison distance matrix of pregnant mice. Differences between *Atg16l1*^*fl/fl*^ and *Atg16l1*^*∆IEC*^ were tested with PERMANOVA with 10,000 permutations. FDR represents Benjamini-Hochberg corrected *p* values, and adj.R^2^ represents partial omega squares as effect size in the analysis of variance. d. relative abundances of the top 20 most abundant genera. Unclassified genera and those with low relative abundance are grouped as “others”. Colors represent individual phylum and color gradients represent individual genus within a phylum. e. triangular dot-plot showing significantly changing genera compared to baseline. The triangles show the hues and direction of the effect size (log_2_FC). Color intensity and size represent the magnitude of the effect size and FDR significance of each specific genus, respectively. f. top three unstable genera with significantly increased abundances in any timepoint compared to baseline. g. top three unstable genera with significantly decreased abundances in any timepoint compared to baseline. The longitudinal plots are colored to remark differences in genotype.

The conditional *Atg16l1* knockout mouse line is described in detail elsewhere.^32,34^ In brief, deletion of the exon 1 flanked by loxP sites was achieved by villin-promoter-driven cre-recombinase excision, which targets all major splice variants of the gene. The details and the model were described before.^32,34^ Control female loxed animals (*Atg16l1*^*fl/fl*^) (also termed control animals or WT) were employed as littermate controls to the female villin-cre (+) mice carrying a conditional deletion in the intestinal epithelium (termed *Atg16l1*^*∆IEC*^ or KO). Timed matings were performed using littermate male *Atg16l1*^*fl/fl*^ mice enabling us to study the dynamics of gut microbiota transitioning between nonpregnancy and pregnancy states with collection of fresh stool pellets at baseline BL (day −1), from trimester 3 (at the end of week 3, termed w3, day 18 after timed mating), and from the end of lactation (at the end of week 6, termed w6, day 39 after timed mating) in experiment 1 (Figure 1a) and sampling at baseline BL (day −1), and in trimester 1, 2, and 3 (termed w1, 2, and 3), at day 6, 12 and 18 after timed mating in experiment 2 (Figure 3a). The mice were housed under specific-pathogen-free (SPF) conditions in individually ventilated cages (IVCs) under a 12 h light–dark cycle. Standard control diet (Sniff, V1534–300, Soest, Germany) and water were given to the animals ad libitum before, during, and after pregnancy, and environmental conditions were maintained at 21°C ±2°C with 60% ± 5% humidity. All animal experiments were approved by the local animal safety review board of the federal ministry of Schleswig-Holstein and conducted according to national and international laws and policies (V 241–69128/2016 (3–1/17) and V 242-7224.121-33). Mice were sacrificed by cervical dislocation. For RNA extraction, tissues were snap-frozen in liquid nitrogen and stored at −80°C. The fecal pellets sampled for reported time points were stored at −80°C until DNA extraction, lipocalin-2 quantification, and metabolomics. Serum was terminally collected and stored at −80°C for metabolomics and Cytokine ELISA.

### Lipocalin-2 quantification

Intestinal mucosal inflammation was monitored through the levels of Lipocalin-2 in stool. The fecal pellets sampled for the reported time points were stored at −80°C until DNA extraction and Lipocalin-2 quantification. The Lipocalin-2 concentration in stool samples was assessed using the Mouse Lipocalin-2/NGAL DuoSet ELISA commercial kit (DY1857, R&D Systems, Minneapolis, USA) specifically designed for mouse Lipocalin-2. The procedure followed the manufacturer’s instructions, with samples diluted at a ratio of 1:10 and added to the plate. Optical density readings were obtained using a plate reader (Infinite M200 Pro, Tecan Männedorf, Switzerland) at wavelengths of 450 and 540 nm. Lipocalin-2 concentrations were then normalized to the weight of feces in each sample.

### RNA isolation and quantitative real-time PCR analysis

For extracting tissue-specific RNA, tissue samples were mixed with 350–600 µl lysis buffer and were disrupted by rapid agitation in a TissueLyser system. Following this, the lysate was processed using the described protocol of the RNeasy Mini kit (Qiagen, Germany). Finally, the purified RNA was eluted and stored at −80°C till further usage. Expressions of candidate genes (TNFA, CXCL1, and IL-10) were normalized using housekeeping genes (GAPDH/β-actin) and an unpaired nonparametric Mann–Whitney test was performed to test genotype-specific differences between nulliparous and pregnant mice as described.^32^

### Stool DNA isolation and 16S rRNA amplicon and shotgun sequencing

DNA from stool samples from the main (87 samples) and validation (56 samples) experiments were isolated using the DNeasy PowerSoil Pro Kit (Qiagen) following the manufacturer’s protocol. Extracted DNA was eluted from the spin filter silica membrane with 100 µl of elution buffer and stored at −80°C.

The V3-V4 region of the 16S gene was amplified using the dual-barcoded primers 341F (GTGCCAGCMGCCGCGGTAA) and 806 R (GGACTACHVGGGTWTCTAAT). Each primer contained additional sequences for a 12 base Golay barcode, an Illumina adaptor, and a linker sequence.^35^ PCR was performed using the Phusion Hot Start Flex 2X Master Mix (NEB) in a GeneAmp PCR system 9700 (Applied Biosystems) and the following program [98°C for 3 min, 25× (98°C for 20 s, 55°C for 30 s, 72°C for 45 s), 72°C for 10 min, hold at 4°C]. Performance of the PCR reactions was checked using agarose gel electrophoresis. Normalization was performed using the SequalPrep Normalization Plate Kit (Thermo Fisher Scientific, Darmstadt, Germany) following the manufacturer’s instructions. Equal volumes of SequalPrep-normalized amplicons were pooled and then sequenced on an Illumina MiSeq (2 × 300 nt). Shot-gun metagenomics sequencing was carried out on DNA extracts obtained from stool samples from the main (72 samples) experiment. DNA libraries were generated using the Illumina DNA Prep kit following the manufacturer’s instructions. Libraries were then pooled and sequenced on an Illumina NovaSeq 6000 platform with 2 × 150 bp. On average, the samples yielded 8.8 ± 2.7 (mean ± SD) million reads. Both 16S amplicon sequencing and shot-gun sequencing were performed at the Competence Centre for Genomic Analysis (Kiel).

### 16S rRNA amplicon sequencing data preprocessing

MiSeq 16S amplicon sequence data were processed using the DADA2 workflow^36^ (https://benjjneb.github.io/dada2/bigdata.html), resulting in an abundance of tables of amplicon sequence variants (ASVs). Taxonomy was assigned using the Bayesian classifier provided in DADA2 and using the Silva rRNA database v.138^37^). Samples with >5000 reads were retained for analyses. After this step, the 16S rRNA gene amplicon sequencing of the main and validation experiments retained on average 20 K ± 8 K and 10 K ± 4 K (mean ± SD) reads per sample, respectively.

ASVs not assigned to Bacteria or annotated as Mitochondria, Chloroplast, or Eukaryote were removed. DADA2 outputs were combined into a single object, and phyloseq R (1.40.0)^38^ package was used for downstream analyses.

### Shot-gun metagenomics data preprocessing

Shot-gun reads were processed using an in-house established workflow from our institute (https://github.com/ikmb/TOFU-MAaPO) that relies on BBtools and bowtie2 for mapping and host decontamination and bioBakery tools (MetaPhlAn 4.0 and HUMaN 3.6) for taxonomic and functional potential profiling. Raw reads were preprocessed with bbduk.sh to trim reads and remove adapter sequences, specifically Illumina Nextera adapters, using the following parameters: ktrim = r, k = 23, mink = 11, hdist = 1, minlength = 50, tpe, and tbo. Subsequently, bbduk.sh was used again to filter out potential sequencing artifacts and PhiX reads with parameters: k = 31, ref = artifacts, phix, ordered, cardinality, and minlength = 50.

For host decontamination, we aligned all reads to the mouse genome (GRCm39) using Bowtie2. Reads aligning to the host genome were discarded, while unaligned reads were retained for further analysis. The Bowtie2 alignment was performed with the parameters: –met-stderr, –no-unal, –sensitive, and – end-to-end. The average depth of sequencing of our samples after this step was 2.6 Gb ± 0.7 Gb, and only samples containing >1 Gbp after trimming and host decontamination were kept in the analysis.

Taxonomic features and quantification of microbial communities’ relative abundances on the 72 samples were done by using MetaPhlAn 4.0^39^ with default parameters and the custom SGB database. MetaPhlAn 4.0 relies on unique marker genes of 26,970 species-level genome bins (SGBs) from a collection of highly curated 1.01 M prokaryotic and metagenome-assembled genomes (MAGs). Likewise, functional potential profiling (stratified pathways, gene families, and enzyme categories) in the same samples was computed using HUMAnN 3.6,^40^ with default parameters. All the abundances were transformed to CPMs before any statistical analysis.

### 16S and metagenomics downstream analyses

Most of the univariate and multivariate analyses for the 16S and metagenomics data were done in the R statistical software (v.4.2.1) under phyloseq^38^ (v.1.40.0), vegan^41^ (v.2.6-2), variancePartition^42^(v.1.26.0) and MAasLin2^43^ (v.1.10.0). Within-sample diversity (alpha diversity) was explored by computing diversity indexes (Chao1, Shannon, Simpson, Observed ASVs) on ASV abundance data and finding differences among certain groups (Wilcoxon rank-sum test or Kruskal–Wallis tests were performed). Between-sample diversity (beta diversity) and the differences between maternal genotype groups and time were explored and visualized in a Principal Coordinate Analysis (PCoA) plot. It was quantified as the Aitchison distance on centered-log ratio transformed ASVs counts. Associations of microbiome composition to maternal genotype or other covariates were tested with the implementation of PERMANOVA models (using the adonis2 function from the vegan package). The P and R^2^ values were determined by 10,000 permutations using time. R^2^ was used for computing partial omega squares^44,45^ as an unbiased estimator of effect sizes. *p* values were subject to Benjamini–Hochberg correction.

### Temporal shifts and genotype-specific differential abundance analysis

To detect differences in changes of microbial features (16S rRNA, metagenomics) and pathways (metagenomics) between the maternal genotype over time, we built linear mixed models on center-log transformed abundances in the MaAslin2 package^43^ that included time, maternal genotype, and individual mice as a random variable ($\mathrm{microbialabundances}∼\left(1|\mathrm{mice}\right)+\mathrm{maternal}\text{\textbackslash{}breakgenotype}+\mathrm{time}$). *p* values were corrected for multiple hypothesis testing using the Benjamin–Hochberg procedure, and a false discovery rate <0.05 was defined as a significant threshold. Features (taxa or pathways) appearing in at least 80% of the samples per group were included in the analysis. For the analysis of temporal dynamics, we built linear mixed-models for each maternal genotype (*Atg16l1*^*fl/fl*^ and *Atg16l1*^*∆IEC*^) data: $\mathrm{microbialabundances}∼\left(1|\mathrm{mice}\right)+\mathrm{time}$ to identify genera that significantly changed the abundance at any time point (week 3 or week 6) compared with BL.

### Variance partition analysis

To understand the major sources of variation in our fecal microbiota data at different phylogenetic resolutions (Phylum, Order, Class, Family, Genus, and ASVs), functional categories (Gene Ontology, KEGG Orthology, Enzyme Commission, MetaCyc Reactions), and metabolic pathways (MetaCyc Pathways), we applied variancePartition (v.1.26.0),^42^ which uses linear mixed models to compute the attributable percentage of variation of a feature based on selected covariates. Here, we selected Individual mouse, maternal genotype, time, lipocalin, maternal weight, and number of pups as possible contributors to the variation of the fecal microbiota composition.

### Metabolomics analysis

Metabolites were extracted from serum samples using the following method: 100 µl serum was transferred to 300 µl ice-cold methanol. Samples were mixed for 3 min on a shaker. An aliquot of 20 µl was taken from each sample to prepare a pooled quality control sample. Each sample was spiked with 100 μL 5α-cholestane (1 mm) and 40 μL ribitol (0.2 mg mL-1). After centrifugation (9,600 × g, 2 min, 0°C) the supernatant was transferred to new tubes and evaporated to dryness in a vacuum concentrator without heating (2 h) (Concentrator plus, Agilent). Dried samples were stored at −80°C until metabolite derivatization. Fresh frozen fecal pellets were weighted and extracted with acetonitrile:methanol:water mixture (2:2:1 vol./vol.) to reach a final concentration of 60 mg fecal mass per 1 ml extraction solvent. Each sample was extracted with ice-cold extraction solution and four metal beads (diameter 3.2 mm) and thoroughly mixed for 2 min. Samples were centrifuged (9,600 × g, 2 min, 0°C), and the supernatant was transferred to new tubes and evaporated to dryness in a vacuum concentrator without heating (4 h) (Concentrator plus, Agilent). Dried samples were stored at −80°C until metabolite derivatization.

Metabolite derivatization was performed by adding 80 μL methoxyamine hydrochloride dissolved in pyridine (20 mg·mL-1) to the dried extracts of serum or fecal pellets and incubated for 90 min at 37°C using a thermomixer (BioShake iQ, Analytik Jena) under constant rotation at 1350 rpm. Following the addition of 100 μL *N,O*-Bis(trimethylsilyl)trifluoroacetamide, each extract was vortexed, and incubated for 30 min at 37°C on a thermomixer at 1350 rpm. After a short centrifugation, 100 μL of the supernatant was transferred to a GC-MS vial for GC-MS data acquisition using the same method described in Michellod et al.^46^ Metabolites were assigned using the NIST 2.0 Library and verified by injection of pure compounds under the same chromatographic conditions. Metabolomics data analysis was done in a pairwise comparisons between-sample groups from different treatments using Chemstation AIA exported files with XCMS online (https://xcmsonline.scripps.edu/.) set to default parameter for file import (GC/Single Quad – centWave).

## Data and code availability

The 16S rRNA amplicon sequencing and the shot-gun metagenomics data are accessible through the European Nucleotide Archive (ENA, https://www.ebi.ac.uk/ena accessed on 8th November 2023) under the accession number PRJEB70999. Additional data supporting the findings of this study, as well as all codes used to generate the bioinformatic analyses, are available from the corresponding author and hosted at https://github.com/viclopezag/ATG16L1_IEC_pregnancy.

## Results

### Longitudinal differences of fecal microbiota of Atg16l1^fl/fl^ and Atg16l1^∆IEC^ during pregnancy and lactation periods

To explore the functional impact of ATG16L1-mediated autophagy in the intestinal epithelium on the dynamics of fecal microbiota during pregnancy, we performed 16S rDNA and shotgun sequencing in 15 weeks female littermate villin (V)-cre+; Atg16l1^fl/fl^ (hereafter called Atg16l1^ΔIEC^) or villin (V)-cre^−^ Atg16l1^fl/fl^ (hereafter called Atg16l1^fl/fl^ or WT) mice.^32^ We longitudinally collected three stool samples per mouse (*n* = 14 mice: *Atg16l1*^*fl/f*^ , and *n* = 15 mice: *Atg16l1*^*∆IEC*^) from the day before timed mating (baseline, termed BL, day 0), from trimester 3 (at the end of week 3, termed w3, day 18 after timed mating), and from the end of lactation (at the end of week 6, termed w6, day 39 after timed mating) (Figure 1a). Timed mating is described as day 0 of the experiment, where male mice were put into cages of female mice for three nights (timed pregnancy induction). In the 16S rRNA data, significant differences in α-diversity (within-sample) between *Atg16l1*^*fl/fl*^ and *Atg16l1*^*∆IEC*^ animals were neither observed during pregnancy nor at the end of lactation (Figure 1b, Supp. Figure S1a). In contrast, the microbiome community composition (α-diversity, between-sample), varied significantly between *Atg16l1*^*fl/fl*^ and *Atg16l1*^*∆IEC*^ in the third trimester (w3) compared to BL (representing the third-trimester PERMANOVA on between-sample Aitchison distances, adj.R2 = 0.063, Benjamini–Hochberg corrected *p* = 2.9e^−4^) and recovered until the end of the lactation period (w6) (Figure 1c, Supp. Fig S1B, 1C, and 1D).

At the taxonomic level of the most abundant taxa, we found – as expected^12^ – a significant overall pregnancy-related increase in the mean relative abundances of Firmicutes and a reduction in Bacteroidetes that seem to recuperate after the lactation period in both *Atg16l1*^*fl/fl*^ and *Atg16l1*^*∆IEC*^ mice (Figure 1d, Supplementary Figure S1E). Interestingly, appreciable differences were observed between *Atg16l1*^*fl/fl*^ and *Atg16l1*^*∆IEC*^ mice at w3 for some genera, e.g., a significant decrease in *Lachnospiracea NK4A136 group* and *Lachnospiraceae UCG-001* and a significant increase in *Turicibacter* in *Atg16l1*^*∆IEC*^ mice (Figure 1d).

To further understand the dynamics and stability of certain taxa during pregnancy and lactation, we performed a multivariate analysis using MaAsLin2^43^ using the 16S rRNA data. We built linear mixed models to identify which genera showed a time-dependent significant difference in abundance compared to BL in *Atg16l1*^*fl/fl*^ and *Atg16l1*^*∆IEC*^ mice. We found 42 temporal shifts of genera that increased or decreased significantly in abundance at w3 (trimester 3) and/or w6 (after lactation) compared to BL (FDR < 0.05, Figure 1e, Supplementary Figure S2A and 2B). At the end of pregnancy (w3), 6 genera, belonging to the Actinobacteriota (*Enterorhabdus*) and Firmicutes (*Lachnospiraceae* UCG-001, *Lactobacillaceae* HT002, *Ligilactobacillus*, *Butyricicoccus* and *Lactobacillus*) phyla, significantly increased in both *Atg16l1*^*fl/fl*^ and *Atg16l1*^*∆IEC*^, whereas 4 genera, belonging to Bacteroidota (*Rikenellaceae* RC9 gut group, *Bacteroides*, *Prevotellaceae* UCG-001, and *Parabacteroides*) phyla, significantly decreased in both *Atg16l1*^*fl/fl*^ and *Atg16l1*^*∆IEC*^. The same comparison at the end of the lactation period (w6) showed that 9 genera, mostly belonging to Firmicutes (*Lachnospiraceae* UCG-001, *Lachnospiraceae* FCS020 group, *Candidatus Arthromitus*, *Roseburia*, *Family XIII* UCG-001, *Butyricicoccus*, *Clostridium sp. ASF356*), Campylobacteria (*Helicobacter*), and Deferribacteres (*Mucispirillum*) phyla increased in both *Atg16l1*^*fl/fl*^ and *Atg16l1*^*∆IEC*^, whereas 7 genera, belonging to Firmicutes (*Family XIII AD3011* group, *[Eubacterium] siraeum* group, *[Eubacterium] nodatum* group, *Christensenellaceae R*-7 group, and *Turicibacter*), Proteobacteria (*Parasutterella*), and Patescibacteria (*Candidatus Saccharimonas*) phyla, significantly decreased in both *Atg16l1*^*fl/fl*^ and *Atg16l1*^*∆IEC*^. Although the abundance shift direction (up or down) was similar for numerous genera in both groups, we noted a diminished count of genera, demonstrating a significant temporal shift in *Atg16l1*^*∆IEC*^ mice compared to *Atg16l1*^*fl/fl*^ mice and several genera showed a significant difference between the groups at the timepoints w3 and/or w6 (Figure 1e). The top three most unstable genera by effect size (Log_2_FC) in *Atg16l1*^*fl/fl*^ and *Atg16l1*^*∆IEC*^ animals are represented in Figures 1f and 1g. We could confirm several genera with common longitudinal dynamics by mining shotgun metagenomics data at strain-level resolution (Supplementary Fig S2C and S2D). Specifically, at w3, *Lactobacillus johnsonii*, *Limosilactobacillus reuteri*, and *Ligilactobacillus murinus* significantly increased and *Muribaculum gordoncarteri* and *Muribaculaceae bacterium* Isolate 110 hZI decreased in abundances (upplementary Figure S2E). In summary, the results suggest that loss of functional *Atg16l1* in the intestinal epithelium of mice is associated with a perturbation of pregnancy-induced fecal bacterial community shifts during pregnancy.

### Maternal genotype is the main driver of explainable fecal microbiota variation at the end of pregnancy between Atg16l1^fl/fl^ and Atg16l1^∆IEC^ mice

We next studied the relative contribution of an ATG16L1 deletion in comparison to other experimental factors to overall microbiota variation. To methodically investigate the impact of experimental covariates, we conducted a variance partition analysis^42^ to quantify the extent to which the variation in each taxon can be attributed to differences in specific covariates. This analysis was done using the 16s rRNA data on different phylogenetic levels (Phylum, Class, Order, Family, Genus, and ASVs) and four different comparisons: (1) a complete dataset including samples from all time points to account for time-dependent effects, and on subsets of samples representing the three individual time points, i.e. (2) only baseline, (3) only w3 and (4) only w6. Before building the variance partition models, we identified co-linearity between the different experimental covariates by canonical correlation analysis (Supplementary Figure S3A). Those covariates with a correlation coefficient <0.6 were considered non-colinear and therefore were included in the variance partition models. Variance partition models were built for the respective data sets in the following way: (1) all time points : *Taxa Abundances* ~ *maternal genotype + weeks + number of pups + lipocalin-2*; and (2) each time point subset: *Taxa Abundances ~ maternal genotype + maternal weight + number of pups + lipocalin-2* (Supplementary Figure S3). Fecal lipocalin-2 levels were assessed as a proxy for mucosal inflammation,^47^ which is known to impact microbial community composition. At no timepoint, we found pairwise differences between genotypes or timepoints; however, we decided to keep this variable to account for potential subclinical inflammation at the level of the individual mouse.

**Figure 2.**
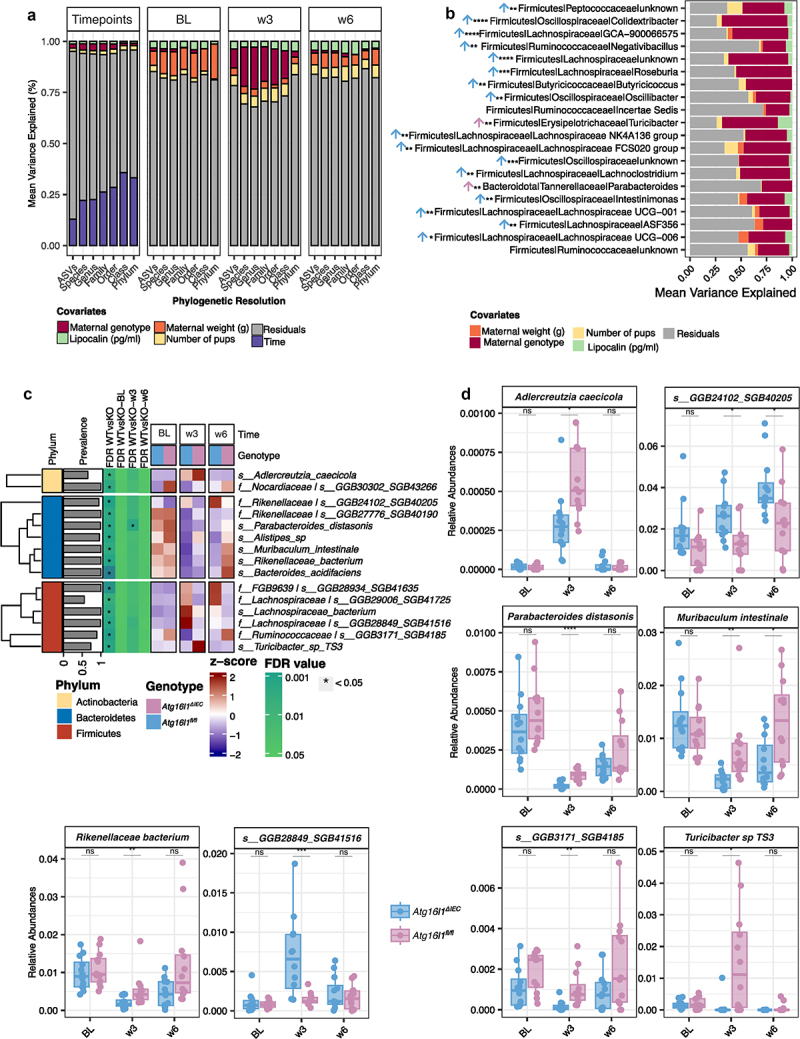
Taxonomic profiling differences of the gut microbiota of *Atg16l1*^*fl/fl*^ and *Atg16l1*^*∆IEC*^ during pregnancy and lactation periods. a. variation partition analysis of different taxa-level abundances from 16S rDNA data. Each color represents the contribution of individual covariates as a source of variation. Each stacked-area bar plot compartment (timepoints, BL, w3, and w6) represents the output of different variance partition models. b. top 20 genera mainly contributing to the variation of the maternal genotype at week 3. Cross-sectional comparisons (*Atg16l1*^*fl/fl*^ vs *Atg16l1*^*∆IEC*^) were performed using the Wilcoxon rank-sum test, and *p* values were corrected by the Benjamini–Hochberg method. *p < 0.05, **p < 0.01, ***p < 0.001, ****p < 0.0001, empty: not significant. The color of the arrow symbol represents the sense of the increased effect of each significant genus (*Atg16l1*^*fl/fl*^, blue or *Atg16l1*^*∆IEC*^, pink). c. heatmap of significant abundance changes in identified species and SGBs found during multivariate comparison of *Atg16l1*^*fl/fl*^ vs *Atg16l1*^*∆IEC*^. for each cell, colors indicate the row-wise z-score of relative abundances, asterisks denote the FDR < 0.05 significance at each cross-sectional comparison and prevalence represents the percentage of non-zero features. Row-wise clusters represent features that belong to the same phylum. d. Box plots showing the relative abundance of selected species and SGBs, the dots represents fecal samples of *Atg16l1*^*fl/fl*^ (n = 12) and *Atg16l1*^*∆IEC*^ (n = 12) pregnant mice per time point.

The complete model could explain around 31% of the total variation (Figure 2A and Supplementary Table S2). Specifically, the main driver of variation of the fecal microbiota across samples was time (approximately 26% in all taxa), followed by maternal genotype (approx. 2.4%), number of pups (approx. 1.92%), and lipocalin-2 (around 1.0%) (Supplementary Table S2). Also, for the time point subsets BL and w6 (at the end of lactation), the maternal genotype had a small (approx. 2%) contribution to the microbial variation; interestingly, the maternal genotype was the main driver of variation in the trimester 3 timepoint (w3) with around 14%, followed by the number of pups (approx. 6%), lipocalin-2 (around 4%) and maternal weight (around 3%) (Supplementary Table S2), in line with the previous observation for the α-diversity analysis. We then examined the top 20 genera that contribute the most to the variation associated with maternal genotype in trimester 3 (Figure 2b). Notably, among the top 20 ranked features, we found a broad spectrum of genera, including Firmicutes, such as *Colidextribacter* (VE = 64%), *Turicibacter* (VE = 55%), *Roseburia* (VE = 54%), *Lachnoclostridium* (VE = 49%), various types of *Lachnospiraceae* (VE = 46%, VE = 40%, VE = 36%, and VE = 30%), and Bacteroidota like the genus *Parabacteroides* (VE = 30%), which together explain most of the genotype-dependent differential abundances between pregnant *Atg16l1*^*fl/fl*^ and *Atg16l1*^*∆IEC*^ mice in trimester 3 (week 3) (full list in Supplementary Tables S3 and S4).

### Species-level differences in the fecal microbiota of pregnant and lactating Atg16l1^fl/fl^ and Atg16l1^∆IEC^ mice

We next investigated species-level (and ASV) differences between the fecal microbiota of *Atg16l1*^*fl/fl*^ and *Atg16l1*^*∆IEC*^ mice using shotgun metagenomics (and 16S rRNA) data. We again performed a multivariate analysis to extract genotype-dependent significant differences in species abundance at the specified time points (BL, w3, and w6). We built four types of linear mixed models in MaAsLin2; a complete model: *bacteria abundances ~ (1|individual mice) + time + maternal genotype* and three models for each time point (BL, w3, and w6): *bacteria abundances ~ maternal genotype*, accounting for the maternal genotype and in the complete model for the effect of time and individual trajectories of mice. We found 15 metagenomic features that were significantly different between *Atg16l1*^*fl/fl*^ *and Atg16l1*^*∆IEC*^. From these, only eight were identified at species level, and seven were identified as species-level genome bins (SGBs). Specifically, we found six Firmicutes (*Turicibacter sp. TS3*, and four SGBs belonging to *Lachnospiraceae* and *Ruminoccaceae* families), seven Bacteroidetes (*Parabacteroides distanosis*, *Alistipes sp*., *Muribaculum intestinale*, *Bacteroides acidifaciens*, and four SGBs belonging to *Rikenellaceae* family), and two Actinobacteria (*Adlercreutzia caecicola* and 1 SGB belonging to *Nocardiaceae* family). Like in the genus-level analysis, most of the species and SGBs had similar longitudinal overall abundance patterns (up or down) between genotypes, but many of the taxa differed significantly in relative abundance at the end of pregnancy (w3) (Figure 2c and 2d). For instance, *Adlercreutzia caecicola, Muribaculum intestinale, Parabacteroides distanosis, Turicibacter sp. TS3*, and *Rikenellaceae* bacterium exhibited a notable rise in relative abundance in *Atg16l1*^*∆IEC*^ compared to *Atg16l1*^*fl/fl*^ at w3. In contrast, certain SGBs associated with the *Rikenellaceae* family (SGB40205) and *Lachnospiraceae* family (SGB41516) displayed the opposite pattern. We could confirm that similar taxa profiles were present at the ASV level (Supplementary Figure S4A and B).

### Inference of functional differences in the fecal microbiota of Atg16l1^∆IEC^ and Atg16l1^fl/fl^ mice from shotgun metagenomics

**Figure 3.**
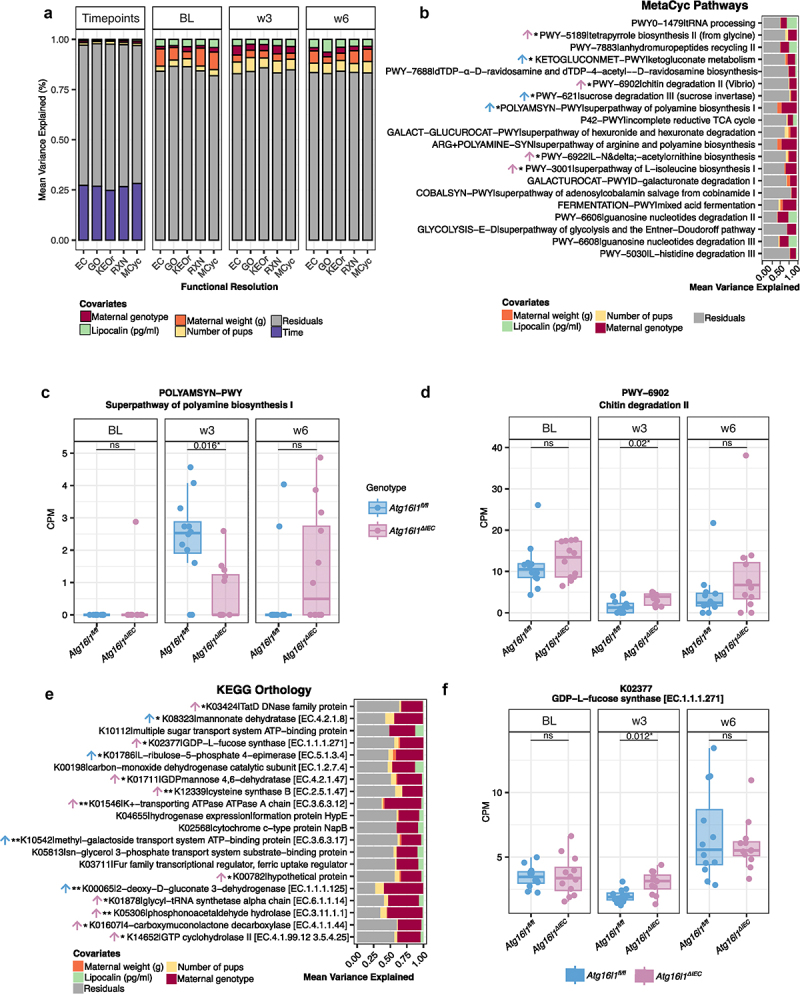
Functional potential predictions of the gut microbiota of *Atg16l1*^*fl/fl*^ and *Atg16l1*^*∆IEC*^ during pregnancy and lactation periods. a. variation partitioning analysis of different functional categories abundances from metagenomics data. Each color represents the contribution of individual covariates as a source of variation. Each stacked-area bar plot compartment (timepoints, BL, w3, and w6) represents the output of different variance partition models. b. top 20 MetaCyc pathways mainly contributing to the variation of the maternal genotype at week 3. c. boxplot of the abundances of polyamine biosynthesis pathway. d. Boxplot of the abundances of chitin degradation pathway. Boxplots are colored to remark differences in genotype. e. top 20 KEGG orthologs that most contribute to the variation of maternal genotype at week 3. The arrow symbol and colors represent the sense of the increased effect of each significant genus (*Atg16l1*^*fl/fl*^, blue or *Atg16l1*^*∆IEC*^, pink). f. box plot of the K02377 gene family, GDP-L fucose synthase. Boxplots are colored to remark differences in genotype. Cross-sectional comparisons (*Atg16l1*^*fl/fl*^ , n = 12 mice vs *Atg16l1*^*∆IEC*^, n = 12 mice) were performed using the Wilcoxon rank-sum test and *p* values were corrected by the Benjamini–Hochberg method. *p < 0.05, **p < 0.01, ***p < 0.001, ****p < 0.0001, empty: not significant.

Having demonstrated that the genetic deletion of ATG16L1 in the intestinal epithelium in mice is associated with altered trajectories of fecal microbiota changes during and after pregnancy, we next aimed to understand the underlying functional consequences using metagenomic pathway profiling. We first inferred the presence and abundance of microbial community functions using the HUMAnN 3.6 pipeline^40^ from metagenomics data. A total of 494,799 gene families and 324 metabolic pathways were detected. Gene families were regrouped into 4 functional categories: KEGG Orthogroups (KEOrs), Level-4 enzyme commission (EC) categories, Gene Ontology (GO), and MetaCyc Reactions (RXN). Similarly, metabolic pathways were classified into four different superclasses and subclasses according to MetaCyc database.^48,49^ The contribution of different experimental covariates to the functional potential of the fecal microbiota was again analyzed by variance partition analysis^42^ with the same data subsets as in the taxonomical analyses. The complete model explained around 30% of the total variation (Figure 3a, detailed values for each individual category in Supplementary Table S6), with high concordance across the four functional categories (KEOr, EC, GO, and RXN). Like our observations at the taxonomic level, the primary factor influencing the variation in fecal microbiota was time (approx. 26% in all categories), succeeded by the number of pups (around 1.3%), maternal genotype (around 1%), and lipocalin-2 (around 0.7%) (Supplementary Table S6). The percentage of variance explained by the maternal genotype to around 4% at week 3 compared to BL (approx. 1.7%), week 6 (approx. 2%) (Figure 3A and Supplementary Table S6). For all functional levels tested, the increase, however, was less pronounced compared to the taxonomical analysis.

Interestingly, we found strong variation (variance explained >20%) in the MetaCyc pathways associated with maternal genotype at week 3 (Figure 3b and Supplementary Table S7). The alterations were positioned in key metabolic pathways, e.g. Amine and Polyamine biosynthesis (POLYAMSYN-PWY: superpathway of polyamine biosynthesis I [VE = 45%, FDR < 0.05] and ARG+POLYAMINE-SYN: superpathway of arginine and polyamine biosynthesis [VE = 45%, FDR < 0.05]) (Supplementary Table S7, Supplementary Table S8).

A formal test for differential abundance (FDR < 0.05) between *Atg16l1*^*fl/fl*^ and *Atg16l1*^*∆IEC*^ animals for each pathway (see methods for details), verified seven of the top 20 inferred pathways. Notably, polyamine biosynthesis functions (ARG+POLYAMINE and POLYAMSYN-PWY) emerged as a salient diminished function in *Atg16l1*^*∆IEC*^ mice at week 3, although the inferred low abundance is based on Metacyc models^48,49^ and does not exclude the presence of individual reactions of this broad pathway, with the lowest FDR recorded at 0.016 (Figure 3c, Supplementary Table S8). Furthermore, we also observed a significant reduction of the term biosynthesis of the nucleotide sugar dTDP-α-D-ravidosamine (PWY-7688), ketogluconate metabolism (KETOGLUCONMET-PWY), and sucrose degradation III (PWY-621) in *Atg16l1*^*∆IEC*^ mice compared to *Atg16l1*^*fl/fl*^, whereas we observed significantly increased abundances on amino acid biosynthesis (L-isoleucine and acetylornithine), chitin degradation (PWY-6902) and tetrapyrrole biosynthesis (PWY-5189). On the KEGG level (Figure 3d and Supplementary Table S9), terms (KEOrs) associated with genotype-dependent variation at week 3 included, e.g., pentose and glucuronate interconversions (K00065: *kduD*; 2-deoxy-D-gluconate 3-dehydrogenase [VE = 59%, FDR < 0.01], and K08323: *rspA*; mannonate dehydratase [VE = 43%, FDR < 0.05]), and KEOrs associated to Amino sugar and nucleotide sugar metabolism (K01711: *gmd*; GDPmannose 4,6-dehydratase [VE = 37%, FDR < 0.05], and K02377: *fcl*; GDP-L-fucose synthase [VE = 35%, FDR < 0.05]). Again, a formal test for differential abundance of the top 20 KEOrs, confirmed 14 to be significantly different between *Atg16l1*^*fl/fl*^ and *Atg16l1*^*∆IEC*^ mice at the timepoint w3 (FDR < 0.05) (Figure 3e, f and Supplementary Table S10). This included KEOrs from the phosphonate and phosphinate metabolism (K05306: *phnX*; phosphonoacetaldehyde hydrolase) and the two-component system (K01546: *kdpA*; K^+^-transporting ATPase A chain) and the Amino sugar and nucleotide sugar metabolism (K01711: *gmd*; GDP mannose 4,6-dehydratase, and K02377: *fcl*; GDP-L-fucose synthase), which were confirmed as significantly increased gene families in *Atg16l1*^*∆IEC*^ mice at week 3. Most significantly, decreased abundances on KEOrs were associated with pentose and glucuronate interconversions (K00065: *kduD*; 2-deoxy-D-gluconate 3-dehydrogenase, and K08323: *rspA*; mannonate dehydratase), followed by a KEOr of ABC transporter (K10542: *mglA*; methyl-galactoside transport system ATP-binding protein), and Pentose phosphate pathway (K01786: L-ribulose-5-phosphate 4-epimerase).

### A validation experiment recapitulates fecal microbiota differences between Atg16l1^∆IEC^ and Atg16l1^fl/fl^ in trimester 3

**Figure 4.**
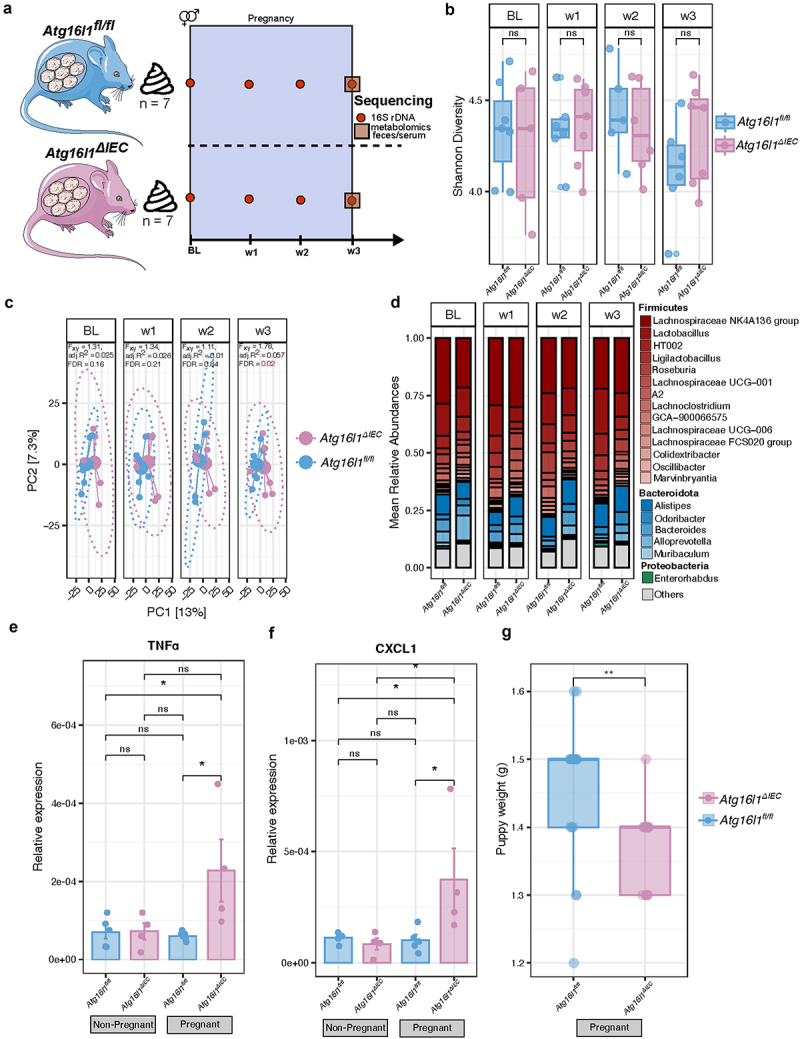
Gut microbiota and cytokine expression characterization of *Atg16l1*^*fl/fl*^ and *Atg16l1*^*∆IEC*^ during pregnancy period (independent validation experiment). (a) Study design. Stool samples of *Atg16l1*^*fl/fl*^ (*n* = 7) and *Atg16l1*^*∆IEC*^ (*n* = 7) pregnant mice were collected weekly from BL (before mating) to week 3 (late pregnancy) and submitted to 16S rRNA amplicon sequencing. (b) Alpha diversity analysis of gut microbiota during pregnancy and after lactation. Shannon diversity was computed at the ASV level. (c) Principal coordinate analysis on Aitchison distance matrix of pregnant mice. Differences between *Atg16l1*^*fl/fl*^ and *Atg16l1*^*∆IEC*^ were tested with PERMANOVA with 10,000 permutations. FDR represents Benjamini-Hochberg corrected *p* values, and adj.R^2^ represents partial omega squares as effect size in the analysis of variance. (d) Relative abundances of the top 20 most abundant genera. Unclassified genera and those with low relative abundance are grouped as “others”. Colors represent individual phylum and color gradients represent individual genus within a phylum. (e, f) Expression levels of the cytokines in nulliparous and pregnant *Atg16l1*^*fl/fl*^ and *Atg16l1*^*∆IEC*^ mice (e) *TNFα* and (f) *CXCL1*. (g) Puppy weight. Groups are shown as nulliparous *Atg16l1*^*fl/fl*^, nulliparous *Atg16l1*^*∆IEC*^, pregnant *Atg16l1*^*fl/fl*^ and pregnant *Atg16l1*^*∆IEC*^. Cross-sectional comparisons (*Atg16l1*^*fl/fl*^ vs *Atg16l1*^*∆IEC*^) were performed using the Wilcoxon rank-sum test. *p < 0.05, **p < 0.01, ***p < 0.001, ****p < 0.0001, ns: not significant.

The previous fecal microbiota characterization suggested that the main effect of the genetic ablation of *ATG16L1* in intestinal epithelial cells can be observed at the end of pregnancy (week 3). To confirm this finding, we performed a validation experiment consisting of a weekly collection and sequencing (16S rDNA) of stool samples throughout a timed pregnancy, from BL to w3 (Figure 4a). At the end of pregnancy, mice were sacrificed, and their biological material was used for phenotypic analysis, including pro-inflammatory markers and characterization of pubs. Results were compared to the respective pregnant littermate wildtype *Atg16l1*^*fl/fl*^. During the pregnancy period, no significant differences or trends in changes in within-sample diversity were observed between *Atg16l1*^*fl/fl*^ and *Atg16l1*^*∆IEC*^ (Figure 4b). In addition, between-sample diversity varied between *Atg16l1*^*fl/fl*^ and *Atg16l1*^*∆IEC*^ only at w3 (PERMANOVA on between-sample Aitchison distances, adj. R2 = 0.057, Benjamini–Hochberg corrected *p* = 0.02) (Figure 4c). These results are aligned with the microbial composition we observed in the previous experiment (Fig 1b, and 1c). Exploring the top 20 most abundant genera, we again found an overall pregnancy-related increase in mean relative abundances of Firmicutes and an overall reduction of Bacteroidetes in both *Atg16l1*^*fl/fl*^ and *Atg16l1*^*∆IEC*^ mice (Figure 4d). Although no formally significant differences were observed between *Atg16l1*^*fl/fl*^ and *Atg16l1*^*∆IEC*^ mice at week 3 at the genus level, we observed a clear trend toward a decrease in *Lachnospiracea NK4A136 group*, *Lachnospiraceae UCG-001*, *Lachnoclostridium*, *Lactobacillus*, and *Roseburia* in *Atg16l1*^*∆IEC*^ mice (Figure 4d, Supplementary Figure S5A) that recapitulates the findings in Figure 1d. Interestingly, the differential abundance analysis at the ASV level showed significant differences in ASVs from the Firmicutes and Bacteroidetes phylum between *Atg16l1*^*fl/fl*^ and *Atg16l1*^*∆IEC*^ during pregnancy (Supplementary Figure S5B). Specifically, we saw significant differences at week 3 in two *Lachnospiraceae* family members as *Lachnospiraceae NK4A136 group*, and *Eubacterium xylanophilum group*; and, in the *Eubacterium brachy group*, an *Anaerovoracaceae* family member.

**Figure 5.**
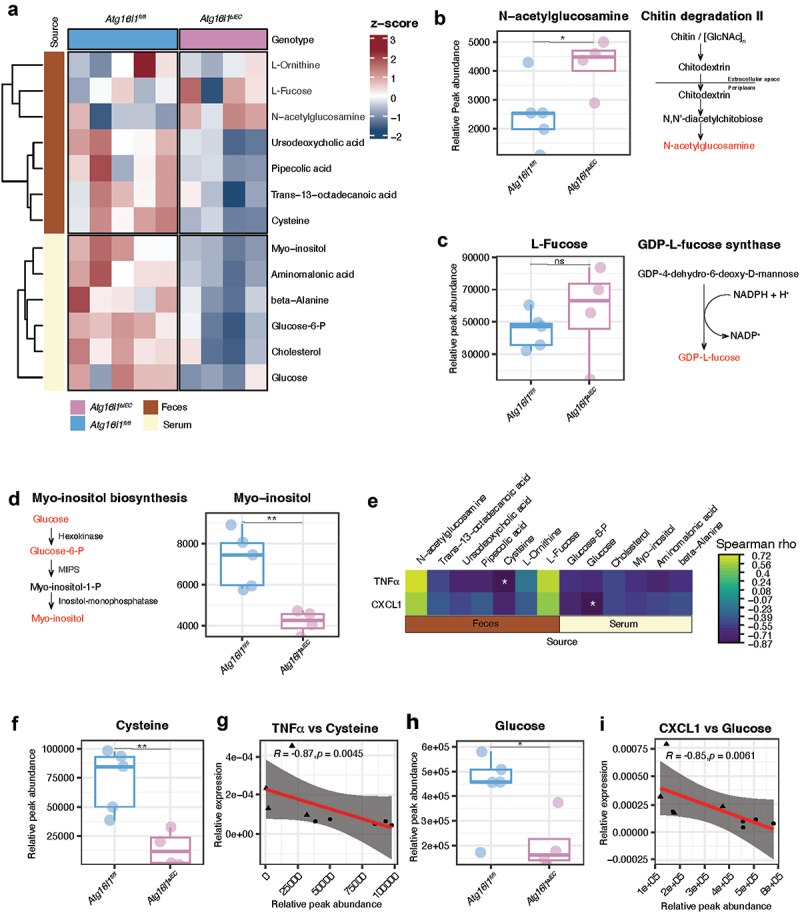
Fecal and serum metabolomics of *Atg16l1*^*fl/fl*^ (*n* = 5) and *Atg16l1*^*∆IEC*^ (*n* = 4) mice at trimester 3 of pregnancy. a. microbiome-specific and significant different metabolites in serum and feces between *Atg16l1*^*fl/fl*^ and *Atg16l1*^*∆IEC*^ mice. b. N-acetyl glucosamine, c. L-Fucose, d. myo-inositol, e. correlation analysis of metabolites and cytokine expression. f, g. cysteine association with tnf-α, h i. glucose association with *CXCL1*.

### Atg16l1 deletion in IECs results in increased inflammatory cytokine secretion in the colonic mucosa of pregnant mice in trimester 3

To understand the physiological and phenotypic effects caused by the absence of *Atg16l1* on intestinal epithelial cells and its consequences for pregnancy, we compared different molecular and physiological parameters between nulliparous *Atg16l1*^*∆IEC*^ (BL) animals and pregnant *Atg16l1*^*∆IEC*^ (week 3) animals (Supplementary Fig S6). As expected, pregnant (week 3) mice showed increased body weight compared to nulliparous (BL) mice within the same genotype: *Atg16l1*^*∆IEC*^ (*p* < 0.05) and *Atg16l1*^*fl/fl*^ (*p* < 0.01) (Supplementary Fig S6A), while there was no detectable difference between the groups at timepoint w3.

Other parameters (liver, spleen weight, and colon length) are presented without significant changes between genotypes as well as timepoints tested (Supplementary Fig 6B, 6C, and 6E). Of note, the cecum weight was significantly higher in pregnant *Atg16l1*^*∆IEC*^ mice compared to pregnant *Atg16l1*^*fl/fl*^ (*p <* 0.05) (Supplementary Fig S6D). Interestingly, we found that pups born from *Atg16l1*^*∆IEC*^ mice were significantly lighter than those that were born from *Atg16l1*^*fl/fl*^ mothers (*p* < 0.01) (Figure 4g). However, no significant differences were found in their length (Supplementary Fig S6F). To further explore the potential impact of changes in microbiota composition during pregnancy on the host, we measured levels of several cytokines in the colonic mucosa of pregnant mice by qPCR. We discovered significantly elevated mRNA levels of pro-inflammatory cytokines, including *Tnf-α* (*p* < 0.05) and *Cxcl1* (*p* < 0.05), in pregnant *Atg16l1*^*∆IEC*^ mice compared to *Atg16l1*^*fl/fl*^ mice (Figure 4(e,f)), suggesting that the deletion of *Atg16l1* in intestinal epithelial cells leads to increased pregnancy-induced intestinal inflammation, potentially originating from the rise of pro-inflammatory bacteria due to the depletion of autophagy and antimicrobial functions in the intestine.

## Metabolomic profiling reveals key metabolic changes in Atg16l1^ΔIEC^ pregnant mice linked to microbial pathways and inflammation

To understand the link between the metabolic potential of the microbial communities and the inflammatory status of the mother, we analyzed fecal and serum metabolites of *Atg16l1*^*fl/fl*^ and *Atg16l1^Δ^*^*IEC*^ pregnant mice at week 3 of pregnancy using gas chromatography-mass spectrometry (GC-MS) (from experiment 2, Figure 4a). These analyses identified five metabolites with significant differences in the feces: N-acetylglucosamine was significantly elevated in *Atg16l1*^*ΔIEC*^ pregnant mice, while urodesoxycholic acid, pipecolinic acid, trans-13-octadecanoic acid, and cysteine were significantly reduced. Similarly, we observed six metabolites that were significantly reduced in the serum of *Atg16l1*^*ΔIEC*^ pregnant mice, including myo-inositol, amino-malonic acid, beta-alanine, glucose-6-P, cholesterol, and glucose (Figure 5a, Supplementary Table S11). Interestingly, we could detect the presence of three metabolites involved in microbial pathways that we previously found to be significantly enriched at the end of pregnancy in *Atg16l1^Δ^*^*IEC*^ mice (Figure 5b, 5c, 5d).

Although we could not reliably detect the presence of polyamines in feces as suggested by metagenomics, we detected L-Ornithine, an important precursor in the polyamine biosynthesis along with agmatine,^50^ in both *Atg16l1*^*fl/fl*^ and *Atg16l1^△^*^*IEC*^ pregnant mice, however with no significant differences (Figure 5a, *p* = 0.725). A nonsignificant trend was observed for L-Fucose in *Atg16l1^Δ^*^*IEC*^ compared to *Atg16l1*^*fl/fl*^ pregnant mice (Figure 5a,5c, *p* = 0.488). This observation is in line with our findings on the high abundance of GDP-L-fucose synthase in *Atg16l1^Δ^*^*IEC*^ mice at week 3 of pregnancy (Figure 3f) and may warrant further investigation. Notably, N-acetylglucosamine (GlcNAc) was found to be significantly higher in *Atg16l1^Δ^*^*IEC*^ compared to *Atg16l1*^*fl/fl*^ mice (Figure 5a, *p* = 0.043). This metabolite is an amino-sugar monosaccharide that is the unit of the polymer chitin^51,52^ and might therefore represent the end product of chitin degradation (Figure 5a,5b), which was also found to be highly abundant in *Atg16l1^Δ^*^*IEC*^ pregnant mice (Figure 3d). GlcNAc is also a precursor of glycosaminoglycans required for epithelial mucin synthesis.^53^

Interestingly, in the sera of *Atg16l1^Δ^*^*IEC*^ mice, we found a significant decrease in glucose (*p* = 0.038), glucose-6-phosphate (*p* = 0.009) and myo-inositol (*p* = 0.004) (Figure 5d), which are all myo-inositol biosynthesis pathway intermediates.^54^ To relate the inflammatory status caused by the absence or presence of *Atg16l1* genotype with the differentially abundant metabolites in feces and serum, we performed a correlation analysis (Figure 5e). From the 13 metabolites, we found two significant associations with cytokine expression of the colonic mucosa: i) a negative association of the expression of *Tnf-α* with Cysteine abundance in feces (Figures 5f, 5g) and ii) a negative association of the expression of *Cxcl1* with Glucose abundance in serum (Figures 5h, 5i).

## Discussion

Pregnancy is associated with a plethora of transient alterations in maternal organ systems, which are all geared toward accommodating the requirements of fetal growth and development. These alterations also comprise structural and compositional changes in the microbiota at different body sites,^8,55^ which are thought to provide an important contribution to the overall adaptation for maximizing reproductive success, e.g. by providing additional energy from nutrient intake.^12^ Relatively little is known about how the host shapes this physiological remodeling of the resident microbial communities, particularly in the gut, and how these changes could contribute to the observed immune alterations during pregnancy.

Intestinal Paneth cells are pivotal players in mucosal immunity and stem cell maintenance and contribute to maintaining a homeostatic intestinal microbiome. We hypothesized that antimicrobial effector functions of the intestinal epithelium could be among the factors that shape the observed pregnancy-associated changes in the intestinal microbiota. Our data from genetically modified mice now provide evidence for the role of the Crohn’s disease risk gene *ATG16L1* in intestinal epithelial cells (IECs) in this process. Hypomorphic or genetically deleted *ATG16l1* in the IEC lineage in mice (and humans) has been shown to cause impaired antimicrobial Paneth and goblet cell architecture,^28^ to increase the propensity for necroptotic cell death^56^ and to impair responses to the protective cytokine Il-22,^32^ which is known to regulate anti-microbial effectors, e.g. defensins in IECs. We have previously demonstrated that under steady state conditions, the microbiota of *Atg16l1*^*∆IEC*^ mice and their respective floxed littermate controls are nevertheless surprisingly similar and that changes in the microbiota only arise from additional perturbations, e.g. when challenging the mice with DSS.^31^ We thus chose this model as an entry point to understand the effect of pregnancy as a physiological perturbation of the intestinal microbiota.

Our mouse model experiment enabled us to study the dynamics of gut microbiota transitioning between nonpregnancy and pregnancy states (week 0 to trimester 3), and then returning to a non-pregnant state (week 3 to week 6). In control and *Atg16l1*^*∆IEC*^ mice, we observed deep changes in β-(between-sample) diversity with time, more specifically, at the timepoint week 3, which corresponds to the third trimester or prepartum. As anticipated, pregnancy itself (i.e. time after conception) was the primary factor influencing changes in the microbiome. This result aligns with previous studies that have observed a significant change in β-diversity in fecal communities during pregnancy, reaching their peak just before delivery.^12^ α-(within sample) diversity indices of fecal microbiota stayed constant during gestational time in our experiments, adding to the controversy on these measures during pregnancy in rodent models and humans. While Koren et al. showed a decrease of within-sample species richness, which contributed to the coining of the term gestational dysbiosis,^12^ other publications did not find temporal changes of α-diversity during pregnancy.^9^ In line with our results, a recent large human study involving approximately 1,500 pregnant Chinese women found no notable variation in fecal α-diversity throughout the entire pregnancy period.^15^ We observed a significant rise in Firmicutes and a decrease in the Bacteroidetes phyla, which confirmed earlier findings in pregnant mice.^9^ Human studies have reported more variable changes already on the phylum level, from a slight increase in *Firmicutes* phylum^57,58^ to dominant changes in *Actinobacteria*, and *Proteobacteria*.^12^ The heterogeneity of maternal fecal microbiota changes in humans has been attributed to recommended or habitual (e.g. increase in carbohydrates) diet changes.^59–62^

Which changes in fecal bacterial communities can be attributed to the absence of functional Atg16l1 in the intestinal epithelium? Starting from a similar baseline composition, we observed a significant difference in beta-diversity between *Atg16l1*^*fl/fl*^ and *Atg16l1^∆IEC^* littermate female mice by the end of the third trimester (day 18 post-mating). This difference is nearly normalized by the end of lactation (week 6), suggesting that the maternal genotype plays a crucial role in driving the observed transient divergance in fecal microbiota. Interestingly, we show that the control (i.e. *Atg16l1*^*fl/fl*^) mice had more systematic temporal changes in individual genera compared to *Atg16l1*^*∆IEC*^ mice. It is well known that *Atg16l1^∆IEC^* mice display morphological and functional abnormalities in the secretory Paneth and Goblet cells.^28,34^ We have previously reported that the impact on microbial communities at steady state is subtle but is unmasked under additional inflammatory signals, such as ER stress.^31^ It can be speculated that normal epithelial secretory functions, including mucus and defensin secretion, may orchestrate microbial dynamics at the end of pregnancy, similar to how defensins regulate microbiota succession in the female reproductive tract during gestation.^63,64^

The main taxonomical differences between *Atg16l1*^*fl/fl*^ and *Atg16l1*^*∆IEC*^ mice comprised members of the *Lachnospiraceae*, *Bacteroidaceae*, *Ruminnococcaceae*, and *Muribaculaceae* families. Specifically, we found a marked reduction of the Short-Chain Fatty Acid (SCFA) producing *Lachnospiraceae* family members like *Roseburia*, *Lachnospiraceae UCG-001*, *Lachnospiraceae NK4A136*, *Lachnoclostridium*, *Lachnospiraceae UCG-006*, and *Lachnospiraceae FCS020 group* and an increase in bacteria like *Turicibacter sp*, *Parabacteroides distasonis*, *Bacteroides acidifaciens*, *Muribaculum instestinale*, *Adlercreutzia caecicola*, and *Ruminococcus sp* in *Atg16l1*^*∆IEC*^ mice during pregnancy. The same taxonomical abundance pattern was found for *Lachnospiraceae*, *Bacteroides ovatus*, and *Parabacteroides* in male mice carrying the *Atg16l1* T300A polymorphism before and after Dextran Sulfate Sodium-induced inflammation, and stool transplantation of active CD patients into germ-free mice.^65^ Interestingly, many taxa that we found as increased or decreased in *Atg16l1*^*∆IEC*^ pregnant mice in trimester 3 have been previously described to have a role in intestinal inflammation, specifically in Crohn’s disease. For instance, a significant reduction in the SCFA-producing bacteria *Roseburia* has been a consistent pattern of CD,^66–69^ while the role of other members of the *Lachnospiraceae* family is more controversial.^70^ Of note, *Lachnospiraceae UCG-001*, the same genus that we found reduced in *Atg16l1*^*∆IEC*^, has been negatively associated with CD in a recent study.^71^ *Bacteroides acidifaciens* has been characterized as a symbiotic organism that inhibits obesity, enhances insulin sensitivity in mice,^72^ which, however, was also associated to gut inflammation in mice models of colitis.^73,74^ *Parabacteroides*, which had a higher abundance in *Atg16l1*^*∆IEC*^ pregnant mice at week 3 compared to controls, were found to be highly abundant in early CD patients,^66^ and specifically *Parabacteroides distasonis* has been identified in intestinal microlesions of a severe CD patient.^75^ The role of *Turicibacter* and *Adlercreutzia* on gut inflammation is less clear. *Turicibacter sanguinis* has been isolated from a febrile patient with acute appendicitis.^76^ In a mouse model with depletion of CD8+ T cells, *Turicibacter* was found to increase in abundance,^77^ while it was found low in mice lacking TNF expression prior colitis induction.^78^ In contrast to our results, Adlercreutzia spp. has been found at lower abundance in CD patients and in relatives of patients with CD.^79,80^ These discrepancies could be associated with the fact that *Adlercreutzia caecicola*, the species we found increased in *Atg16l1*^*∆IEC*^ mice at the end of pregnancy, was originally isolated from the cecal content of male C57BL/6 mice.^81^ Further studies on *Turicibacter* and *Adlercreutzia* genus are necessary to elucidate their role in IBD and gut inflammation.

Several of the discussed taxa have also been implicated in gestation and pregnancy outcomes. In this context, SCFA-producing taxa like, e.g. butyrate-producing *Roseburia* – which we found reduced in pregnant *Atg16l1*^*∆IEC*^ mice – are also found to be reduced or in women with recurrent miscarriage^82,83^ or in obese pregnant women,^84^ where obesity can either result in short-term or long-term complications for both mother and fetus. *Roseburia intestinalis* has been shown to protect against intrahepatic cholestasis during pregnancy (ICP) by restoring intestinal barrier function, reducing Th17 cells, and increasing Treg cells in ICP rats.^85^ This modulation of immune cell types, crucial for fetal tolerance, mirrors findings in TNBS-induced colitis in mice, where R. intestinalis increased anti-inflammatory IL-10 and decreased pro-inflammatory IL-17.^68,86^

Shotgun metagenomics revealed that gut microbiota alterations in *Atg16l1*^*∆IEC*^ mice at the end of pregnancy may affect their metabolic capacity, e.g. a reduced capacity to biosynthesize polyamines, metabolize acidic polysaccharides like keto-gluconate and degradate sucrose. Although little is known about the role of microbial-derived polyamines like putrescine and spermidine, recent studies have shown that dysbiosis in gut microbiota can trigger imbalances in polyamine metabolism, which may favor metabolic-related diseases.^87^ In fact, spermidine has been shown to exert a protective role against experimental colitis in mice. Supplementation with spermidine suppressed colitis and reduced colitis-associated carcinogenesis.^88^ We could not validate this finding in our fecal metabolomic data, which emphasizes the gap between the inferred metabolic potential and metabolite composition. Metagenomic analysis also suggested that *Atg16l1*^*∆IEC*^ mice may have a higher capacity to produce fucosylated oligosaccharides (via GDP-L-fucose synthase), with this function also found in fucose-carrier *Bacteroides* species of Crohn’s disease patients.^89^ Metabolomic profiling only revealed a suggestive trend of fecal L-Fucose in *Atg16l1*^*∆IEC*^ mice at trimester 3, which may warrant further investigation due to the important role of fucosylation as a post-translational protein modification in Crohn’s disease and colorectal cancer.^26,90–92^

We found consistent evidence for an increase in chitin-degradation in *Atg16l1^∆IEC^* pregnant mice in trimester 3 (day 18 after timed mating), both by higher abundance of the pathway in metagenomic data, as well as increased levels of its metabolite N-acetylglucosamine in the feces of these mice. Chitin, a polymer of β-1, 4 N-acetylglucosamine (GlcNAc), represents a highly abundant polysaccharide produced by fungi, insects, and crustaceans. Chitin has diverse immunoregulatory functions^93^ and chitin microparticles have been suggested as experimental treatment in IBD .^94^ Of note, post-translational modification of proteins by the breakdown product of chitin, *N*-acetylglucosamine, termed O-GlcNAcylation, promotes intestinal inflammation and is enhanced in the inflamed intestine of CD patients.^95^ During pregnancy, increased O-GlcNAcylation has been shown to impact placental functions in women with hyperglycemia during pregnancy.^96^ However, whether and how enhanced chitin degradation is involved in the phenotype of *Atg16l1*^*∆IEC*^ pregnant mice need further investigation.

These changes were associated with a spontaneous inflammatory response in the colonic mucosa of *Atg16l1*^*∆IEC*^ mice at trimester 3 as evidenced by elevated mRNA levels of *Tnfa* and *Cxcl1* and decreased weight of offspring compared to pregnant control mice. Importantly, the observed subtle inflammatory response occurred in the colon and well outside the time window of the previously reported spontaneous inflammation in the mouse strain, which had an onset around the age of 35 weeks.^31^ The serum metabolomics analysis underscores the systemic effects of the deletion of ATG16L1 in the intestinal epithelium in the third trimester of pregnancy. Whereas it is known that during a healthy pregnancy glucose and cholesterol levels increase,^97^ *Atg16l1*^*∆IEC*^ mice show significantly reduced levels of glucose, glucose-6-phosphate, and cholesterol in the serum. Normally, maternal metabolism during pregnancy shifts from glucose oxidation to fatty acid oxidation and increases maternal glucose production via enhancing hepatic gluconeogenesis to provide glucose to the developing fetus. Hence, our finding in *Atg16l1*^*∆IEC*^ mice may point toward a potential undersupply to the fetus, which could lead to the observed reduced weight of the offspring. Furthermore, we detected a significant negative correlation between the inflammatory marker *Cxcl1* in the gut and glucose levels in the serum. Of note, this finding resembles a finding in Muc2 -/- mice, which develop spontaneous low-grade intestinal inflammation, and also display lower glucose levels.^98^

In addition, we also found myo-inositol to be reduced in the serum of pregnant *Atg16l1*^*∆IEC*^ mice. Free myo-inositol is synthesized from glucose and glucose-6-phosphate, which also were significantly reduced in pregnant *Atg16l1*^*∆IEC*^ mice. Myo-inositol is known to mediate signal transduction in response to several hormones, neurotransmitters, growth factors, and participates in osmoregulation.^99^ Recently, supplementation studies of this metabolite have shown beneficial effects on pregnancy rates, newborn health, and reduction of neural tube defects.^100–102^ In the feces, we detected significant lower levels of Ursodeoxycholic acid (UDCA) in *Atg16l1*^*∆IEC*^ mice compared to their littermate controls in trimester 3. UDCA has been discussed as a promising therapeutic target for IBD due to its cytoprotective and anti-inflammatory actions^103–108^ as well as for colitis-associated cancer via gut microbiome modulation.^109^ UDCA also promotes Treg differentiation and inhibits M1 macrophage polarization in an DSS colitis model.^110^ Thus, it will be interesting to investigate whether UDCA in the gut may also serve as a modulator of the immune system during pregnancy.

As a limitation of this study, we could not establish mechanistic proof that the physiological differences observed between *Atg16l1*^*∆IEC*^ mice and their *Atg16l1*^*fl/fl*^ littermate controls were directly linked to microbial changes. The usual approach of fecal microbial transfer is hampered by the dynamic nature of the observed microbial phenotype. Repeated transfers of T1-T3 microbiota into wildtype pregnant mice would have been required, with procedures such as antibiotic preconditioning and handling of pregnant mice, potentially affecting pregnancy outcomes.

Previous studies have shown that autophagy is essential for mitophagy during the gestation-to-lactation transition in mammary epithelial cells (MECs) in vitro ^111^. However, large-scale GWAS studies in dairy cattle have not identified Atg16l1 or other autophagy-related proteins as key factors in milk production ^112–114^. Our study specifically involves the deletion of Atg16l1 in intestinal epithelial cells, with no direct effects of hypomorphic *ATG16L1* on MECs, as villin (as the driver of the Cre recombinase) is not expressed in the mammary gland. Although work by us and others on hypomorphic Atg16l1 in intestinal epithelial cells ^28,31,32,34^ has mainly shown an effect on the secretory lineage (i.e. Paneth and goblet cells), we cannot rule out indirect effects on milk production, e.g via perturbed nutrient absorption.

Like in all studies on metagenomics in mice, it is important to note that the tool to infer the functional potential of fecal microbiota has been developed mostly using human-derived data.^40^ It is thus crucial to further validate these inferred changes as genomic information on microbial taxa present in the mouse microbiota might be absent from the data bases or differently regulated in mice.^115–117^ Other constraints might be related to the Metacyc-based modeling of Pathway abundance^48,49^ which could underestimate pathway abundance when individual reactions have low counts. This is exemplified by unexpectedly low abundance of the polyamine synthesis pathway and does not imply the absence of polyamine metabolism but rather highlights a limitation in our analytical pipeline – a challenge also observed in other studies using similar methods.^118,119^ Lastly, caution is necessary, when extrapolating our mouse study results in humans, as the physiological effects observed in mice may differ significantly due to species-specific factors. Further investigation is required to validate potential therapeutic targets, such as the supplementation of ursodeoxycholic acid, polyamines, or SCFAs as protective postbiotics. Additional studies are necessary before considering the translation of these findings to pregnant women with IBD.

In summary, our study suggests that genetic impairment of autophagy in IECs may amplify the pro-inflammatory tone at late stages of gestation.^12^ *Atg16l1*^*∆IEC*^ pregnant mice have overall lesser coordinated changes in their fecal microbiota than their wildtype littermate controls. Several of the salient changes present in *Atg16l1*^*∆IEC*^ female mice in trimester 3 resemble features observed in human IBD patients, e.g. high N-acetyl-glucosamine levels. It is thus tempting to speculate that these changes in female IBD patients with genetic variants in autophagy-related genes such as ATG16L1 T300A^26,65,120^ might modulate the threshold of inflammatory relapses around birth. Our results highlight the importance of better understanding host factors contributing to the functional shifts of the intestinal ecosystem in IBD during gestation and after birth. Clearly, it underscores the necessity for larger-scale cohort studies in female IBD patients to capture the individual heterogeneity and distinct sources of variation of host–microbe interactions during pregnancy. We suggest that rationale microbiota-targeted therapies may represent a powerful addition to improve personalized care and pregnancy outcomes in female patients with IBD.

## Ethics approval

All animal experiments were approved by the local animal safety review board of the federal ministry of Schleswig-Holstein and conducted according to national and international laws and policies (V 241–69128/2016 (3–1/17) and V 242-7224.121-33).

## Availability of data and materials

All data generated or analyzed during this study are included in this published article.

## Supplementary Material

Supplemental Material
