# Supplementary figures and images for: Defective Atg16l1 in intestinal epithelial cells links to altered fecal microbiota and metabolic shifts during pregnancy in mice
Source: Gut Microbes. 2024 Dec 2;16(1):2429267. doi: 10.1080/19490976.2024.2429267 (PMC11622647; doi:10.1080/19490976.2024.2429267)

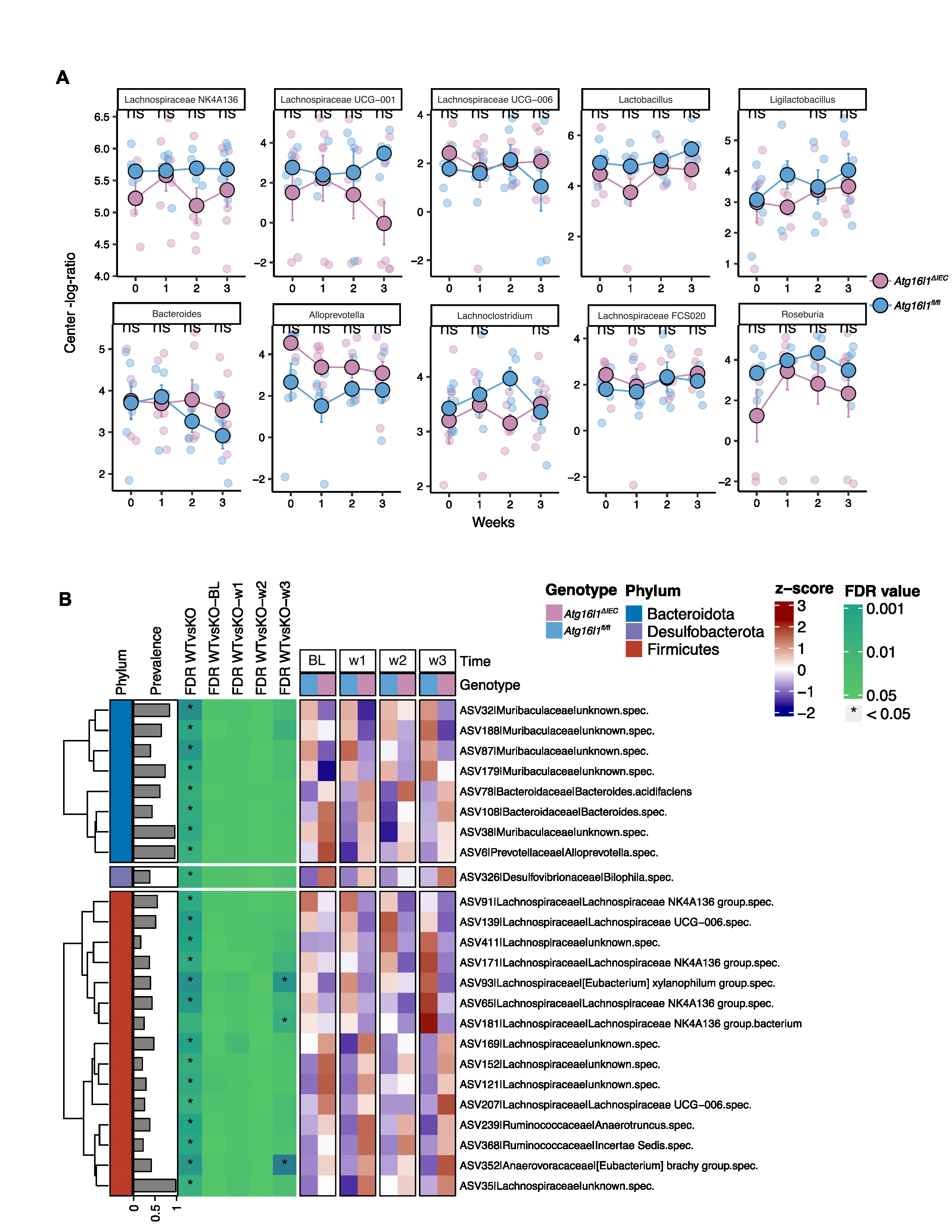

Supplement: Supplemental Material [file KGMI_A_2429267_SM2272.zip › SuppFig5.tiff]

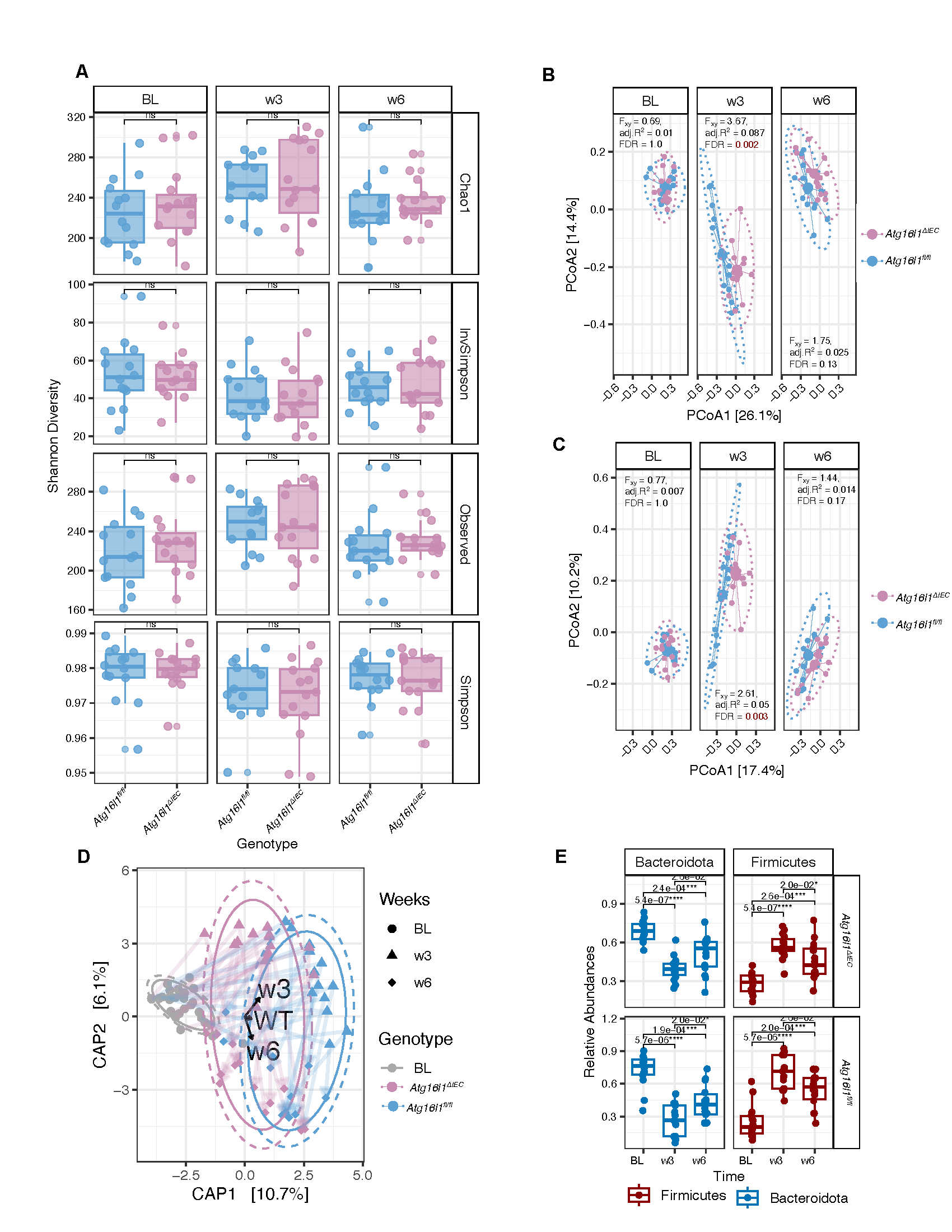

Supplement: Supplemental Material [file KGMI_A_2429267_SM2272.zip › SuppFig1.tiff]

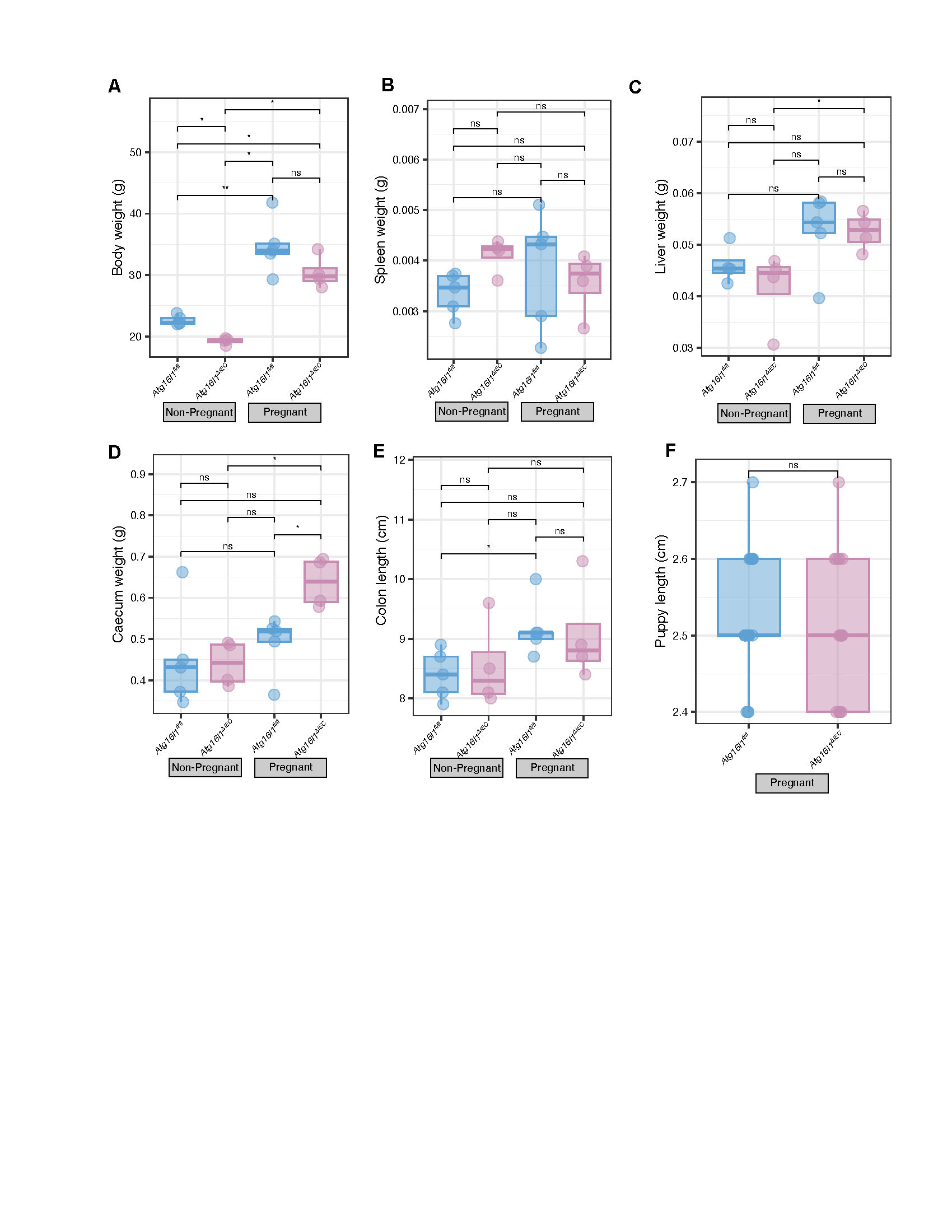

Supplement: Supplemental Material [file KGMI_A_2429267_SM2272.zip › SuppFigure6.tiff]

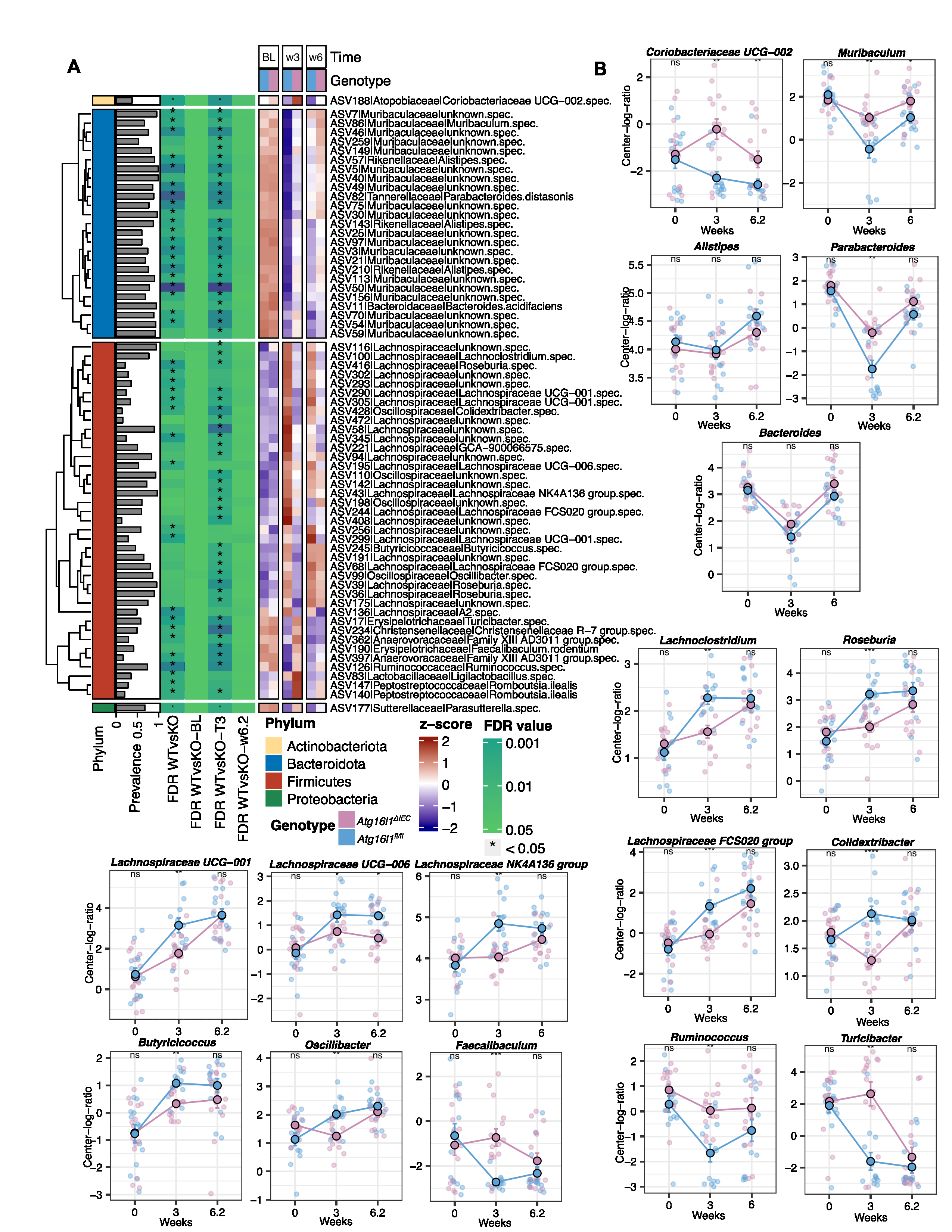

Supplement: Supplemental Material [file KGMI_A_2429267_SM2272.zip › SuppFig4.tiff]

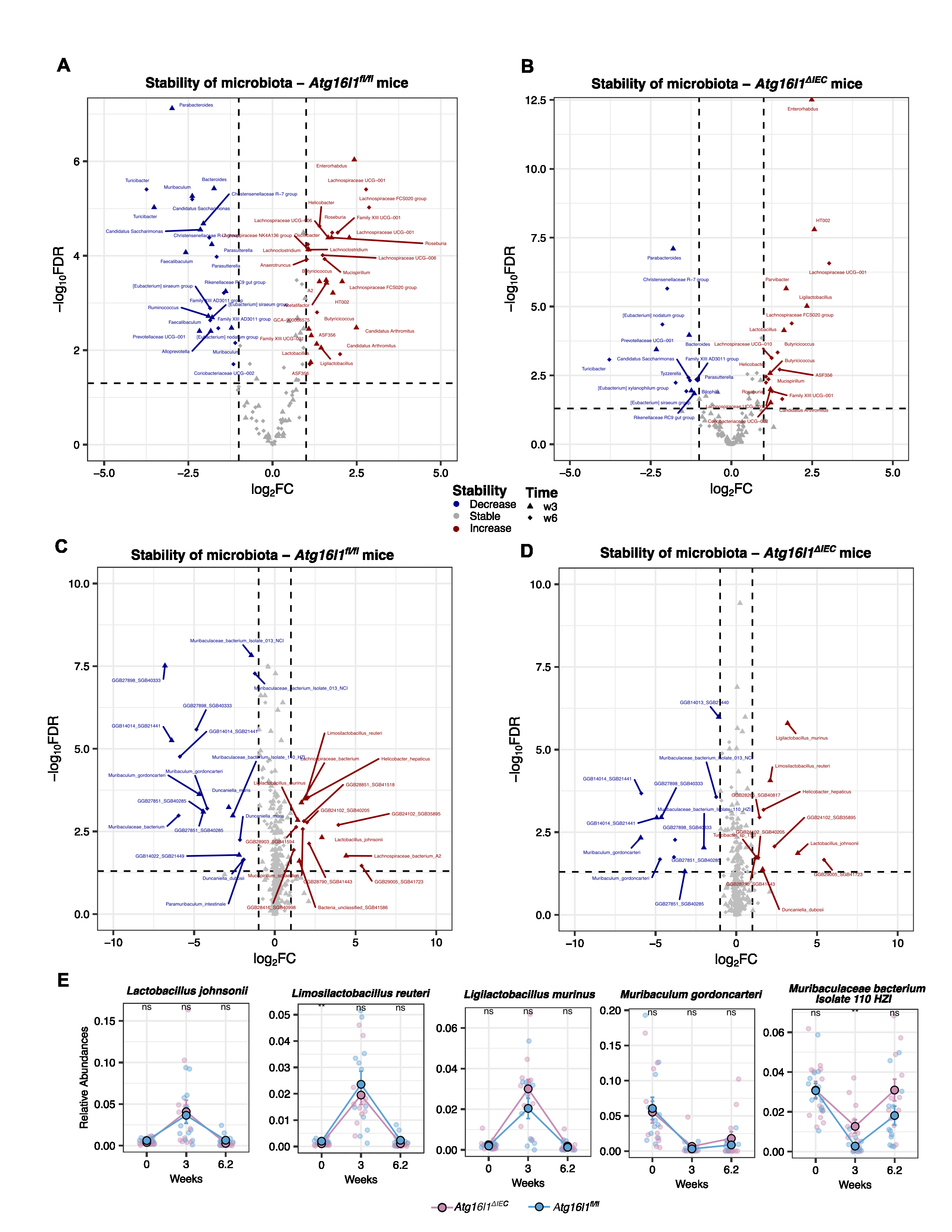

Supplement: Supplemental Material [file KGMI_A_2429267_SM2272.zip › SuppFig2.tiff]

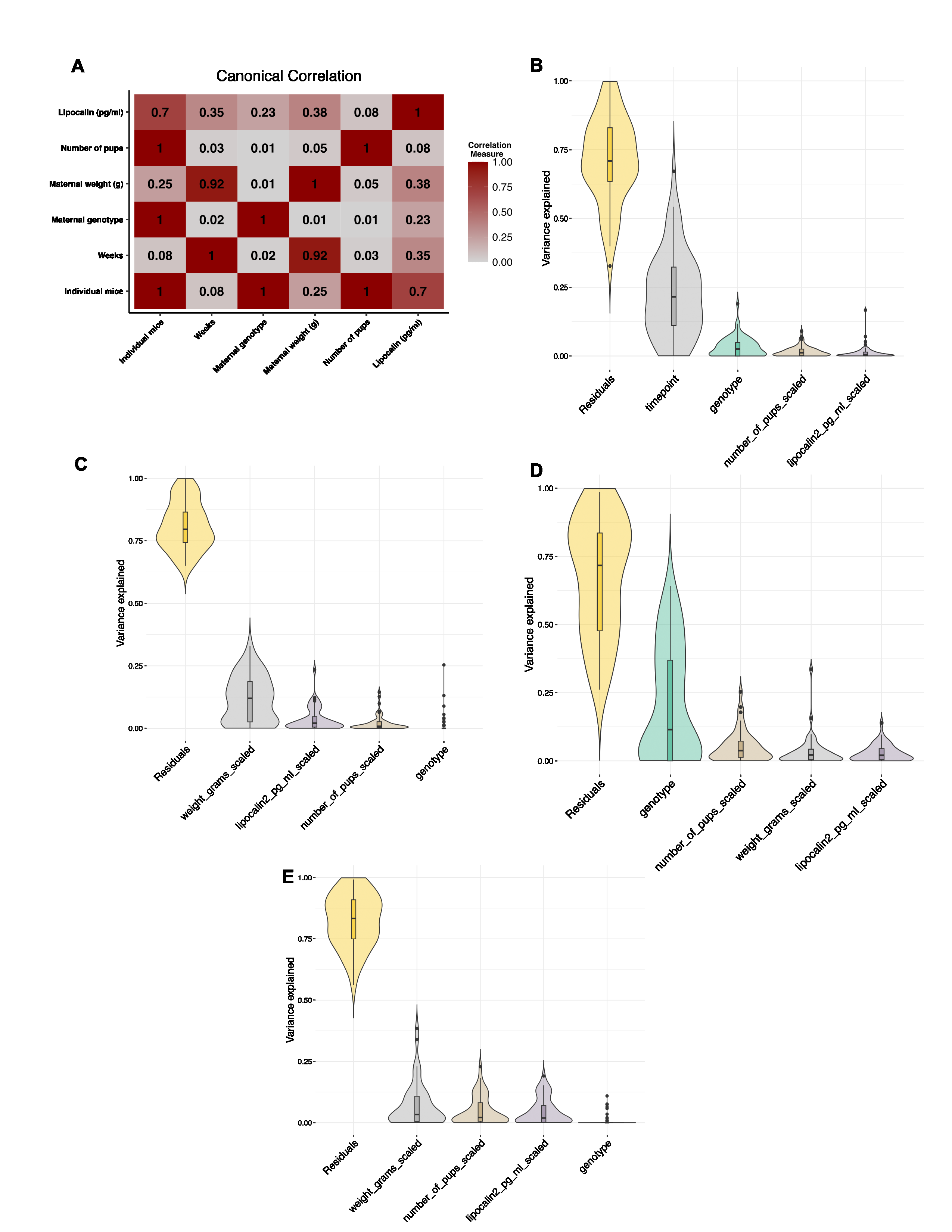

Supplement: Supplemental Material [file KGMI_A_2429267_SM2272.zip › SuppFig3.tiff]
